# Editorial: Emerging roles of circular RNAs in the tumor: functions and potential applications–volume II

**DOI:** 10.3389/fcell.2024.1339274

**Published:** 2024-03-21

**Authors:** Shanchun Guo, Lan Huang, Mingli Liu

**Affiliations:** ^1^ RCMI Cancer Research Center, Department of Chemistry, Xavier University, New Orleans, LA, United States; ^2^ Translational Medicine Center, The First Affiliated Hospital, Zhengzhou, Henan, China; ^3^ Department of Microbiology, Biochemistry and Immunology, Morehouse School of Medicine, Atlanta, GA, United States

**Keywords:** circRNA, tumor, pancreatic cancer, intrahepatic cholangiocarcinoma, liquid biopsy

Circular RNA (circRNA), a class of non-coding RNAs without the 3′cap and 5′poly(A) and linked by covalent bonds, has attracted substantial attention in the field of biological research. The surge in interest has led to a collective understanding of circRNA’s pivotal roles in physiological development and various diseases (Kristensen et al., 2018; Chen and Lu, 2021; Gu et al., 2023; Liu et al., 2023; Pisignano et al., 2023). Recent use of RNA sequencing and bioinformatics methods has discovered a huge number of circRNAs in diverse cell lines and species. Emerging research suggests that many circRNAs exhibit cell-type specific expression and act as either oncogenic triggers or tumor suppressors in the context of cancer (Kristensen et al., 2022). CircRNAs often display distinct expression patterns linked to specific tissues and cancer types and accumulating evidence suggests their clinical significance and utility. Notably, CircRNAs hold great promise as diagnostic, prognostic, and predictive biomarkers, as they are detectable in liquid biopsy samples (Chen and Shan, 2021; Wang et al., 2021; Wen et al., 2021). In a noteworthy development, synthetic circRNAs have been generated to explore their potential as a novel class of mRNA treatments and vaccines (Liu et al., 2022). Recent research findings have shown that some cytoplasmic circRNAs can undergo translation, resulting in detectable peptides, thereby shedding light on the role of circRNAs in cellular physiology and cancer biology (Lei et al., 2020). Despite the inherent challenges associated with detecting, quantifying, and functionally characterizing circRNAs due to their unique nature, recent advancements in high-throughput RNA sequencing and the development of circRNA-specific computational tools have facilitated the creation of cutting-edge methodologies for their identification. Moreover, novel strategies for functional characterization are continually emerging.

The Research Topic “E*merging roles of circular RNAs in the tumor: functions and potential applications- volume II*” focuses on circRNAs’ role and molecular signaling pathways by which they regulate tumor development. We have received numerous manuscripts and finally selected 4 articles in cancer research that were ultimately published.

Seimiya et al. provided a comprehensive summary of 63 representative circRNAs that play pivotal roles in the development of pancreatic cancer. These pathogenic circRNAs have been demonstrated to function as miRNA decoys in pancreatic cancer, contributing to process such as cell proliferation, inhibition of apoptosis, metastasis, chemotherapy resistance, and metabolic reprogramming. Additionally, some circRNAs, such as circFARP1, directly interact with proteins, thus enhancing their stability by preventing ubiquitin-proteasomal degradation. Due to their remarkable stability and specificity, circRNAs hold significant promise as diagnostic biomarkers for cancer. Specifically, circLDLRAD3, circPDAC, hsa_circ_0013587, and circ_001569 have been reported to exhibit abnormal expression in the serum of pancreatic cancer patients with a sensitivity ranging from 0.45 to 0.76 and specificity ranging from 0.70 to 0.90 for diagnosis of pancreatic cancer. Furthermore, the distinct biological roles and molecular properties of circRNAs open up new avenues for their potential application as nucleic acid therapeutic agents in the context of pancreatic cancer.

Sinha et al. harnessed publicly available RNA-sequencing datasets of pancreatic islets to compile a comprehensive catalog of all expressed circRNAs in pancreatic islets. This endeavor culminated in the establishment of the PanCircBase (https://www.pancircbase.net/) database. PanCircBase o offers a wide array of valuable resources, including: 1) Detailed annotation for circRNA in pancreatic islet, encompassing genomic location, host gene, exon information, splice length, sequence, as well as other relevant database identifiers and information regarding cross-species conservation. 2) Distinct primers designed for circRNA PCR analysis, 3) siRNAs that have been used to specifically silence target circRNAs. 4) Information about miRNAs linked to circRNAs, 5) Insights into protein-coding circRNAs and their potential to encode polypeptides. In conclusion, PanCircBase represents an extensive and invaluable online resource for researchers investigating circRNA expression and its potential function in pancreatic cells.

Autophagy plays a pivotal role in maintaining cellular homeostasis and represents a significant molecular process in tumor progression. Numerous studies have demonstrated that autophagy not only aids cancer cells in their survival, but, under specific conditions, can also induce autophagic cell death. In recent years, an increasing number of research studies have unveiled compelling connections between circRNAs and autophagy. In a systematic review and analysis of recent research, Zhou et al. conducted that the circRNA-autophagy axis plays a critical role in various aspects of tumor biology, including tumor cell proliferation, metastasis, invasion, and drug or radiation resistance.

Intrahepatic cholangiocarcinoma (iCCA) represents a complex and diverse condition characterized by a range of etiologies, morphological variations, and clinical outcomes. However, our understanding of its epidemiology and carcinogenesis remains limited. Using high-throughput sequencing techniques, Liang et al. conducted a study investigating the expression patterns of circRNAs in iCCA tissues as well as their corresponding adjacent normal tissues, denoted as (iCCA) and (iCCAP), respectively. The analysis revealed a total of 117 differentially expressed (DE) circRNAs. These DE circRNAs were found to be associated with to several significant GO terms and exhibited enrichment in essential pathways related to the parental transcripts of circRNAs. Furthermore, the researchers constructed two circRNA-mediated ceRNA networks, highlighting the regulatory roles of DE circRNAs in modulating important metabolic pathways through interactions with mRNAs and miRNAs. Their findings shed light on the differential expression of circRNAs in iCCA tissues compared to iCCAP tissues, suggesting that circRNAs may play important roles in the context of cancer.

In conclusion, this Research Topic underscores the multitude of roles and contributions that circRNAs make in human tumors. However, many aspects of circRNA research remain unexplored, such as the functions of peptides encoded by specific circRNAs in tumor contexts and the correlation between circRNA expression and other frequently observed molecular alterations in tumors. Ultimately, it is undeniable that circRNAs represent promising candidates as both cancer biomarkers and therapeutic targets.
